# The effect of exposure to low frequency electromagnetic fields (EMF) as an integral part of the housing system on anxiety-related behaviour, cognition and welfare in two strains of laboratory mouse

**DOI:** 10.1371/journal.pone.0197054

**Published:** 2018-05-17

**Authors:** Oliver Burman, Gerardo Marsella, Angelo Di Clemente, Luigi Cervo

**Affiliations:** 1 School of Life Sciences, University of Lincoln, Lincoln, United Kingdom; 2 IRCCS - Istituto di Ricerche Farmacologiche “Mario Negri”, Animal Care Unit, Milan, Italy; 3 IRCCS - Istituto di Ricerche Farmacologiche “Mario Negri”, Experimental Psychopharmacology, Milan, Italy; University of Queensland, AUSTRALIA

## Abstract

Electromagnetic field (EMF) technology has the potential to improve scientific data capture and welfare assessment by allowing automated data collection from individual cages. However, it is important to determine any impact that a new technology itself may have on animal welfare, and previous studies have found contrasting results of EMF on laboratory rodent anxiety-like behaviour and cognition. We therefore investigated whether there was an effect of low frequency EMF experienced continuously over a six-week period, as an integral part of the animal housing system, on measures of mouse anxiety-related behaviour, cognition and welfare. We housed mice (N = 80) of two strains (BALB/cAnNCrl and C57BL/6NCrl) separately in Individually Ventilated Cages (IVCs) in groups of four, either with the EMF plate turned ‘on’ or ‘off’ (n = 5). Some measures, e.g. food and water utilisation, were collected at regular intervals, whereas measures of anxiety-like behaviour (e.g. open field test) and cognitive performance (novel-object recognition test) were collected only at the end of the study. We found expected strong strain differences in most measures, e.g. latency to leave the starting square in an open field test, with C57BL/6NCrl mice moving away sooner, and interactions between strain and time for those measures recorded at more than one time point, e.g. significant weight gain over time for both strains, but with BALB/cAnNCrl mice weighing more. However, we found no significant effects of treatment (EMF ‘on’/‘off’) for any of the measures collected. These results indicate that, for the measures recorded here, there was no measurable impact on the behaviour and welfare of low frequency EMF exposure experienced continuously over a six-week period. Housing systems that include EMF monitoring technology may therefore be suitable for use without influencing either animal welfare or scientific outcomes.

## Introduction

Electromagnetic field (EMF) technology has the potential to improve scientific data capture and welfare assessment by allowing automated data collection from individual cages, e.g. of animal activity [1], potentially improving the accuracy, detail and range of data that is collected. However, before advancing with a new technology, it is important to determine any influence that the technology itself might have on animal behaviour, and consequently on animal welfare, and the quality of scientific data obtained. Previous studies have found contrasting results of EMF on laboratory rodent anxiety-like behaviour and cognition, either finding no effects on anxiety-like behaviour (e.g. [2] (mice); [3] (rats)), finding changes in anxiety-like behaviour (e.g. [4] (rats); [5] (rats)) or influences on different aspects of cognitive performance (e.g. [6] (rats); [7] (rats)). A possible explanation for these contrasting findings may be due to the different durations and/or frequency of the EMF studied.

Electromagnetic field frequency varies in studies from 50Hz [4] to 2.45GHz [3], with exposure duration ranging from the short-term, e.g. one exposure for 20mins [7], to short-term exposures for medium periods of time, e.g. four hours per day for 25 days [4] to short-term exposures over much longer periods of time, e.g. 60min per day for 120 days [2]. The selection of EMF exposures is partly due to most studies focusing on rodents as a model for potential effects of EMF in humans, but there is increasing interest in the use of EMF-reliant technology for the observation of rodents themselves. For example, in a system to record the activity of rodent models of Huntington’s disease that allows automated monitoring of individual mice housed socially in their home-cages [1]. However, few studies have investigated the effect of continuous EMF exposure when it forms an integral part of the animal housing system itself, i.e. continuous exposure within the home cage. If this technological advance is to prove beneficial when incorporated into animal housing systems, then research is required to identify the potential impact of this technology on the behaviour and welfare of the animals housed within such housing systems.

When it comes to assessing the impact of EMF, various different approaches have been taken. Cognitive effects have been investigated using the water maze (spatial memory: [7]) and social recognition tests [6]. Anxiety-like behaviour has been investigated most commonly using the elevated plus maze and open field tests (e.g. [8]; [9]), with some additional use of the light/dark box (e.g. [4]), the marble-burying and social interaction tests (e.g. [2], and depression-like behaviour investigated using forced swim tests (e.g. [10]). Few studies have investigated spontaneous ‘in-cage’ behavioural changes in response to EMF, a key element of welfare assessment [11]. A more effective approach may therefore be to combine different measures, e.g. spontaneous behaviour, tests of anxiety-related behaviour, and tests of cognitive performance, in order to triangulate an overall interpretation of welfare status (e.g. [12]).

The aim of this study was therefore to investigate the impact of low frequency EMF, experienced continuously over a six week period as an integral part of the animal housing system, on laboratory mouse anxiety-related behaviour and welfare, using a variety of behavioural and cognitive indicators as recommended when defining and implementing protocols for the welfare assessment of laboratory animals [11].

## Materials and methods

### Subjects and housing

For this study we used juvenile (6-8wks of age) female laboratory naïve mice (N = 80) of two commonly used, but behaviourally contrasting [13, 14], strains (C57BL/6NCrl (n = 40) and BALB/cAnNCrl (n = 40)) obtained Specific Pathogen Free from a single external supplier (Charles River, Calco, Italy) arriving at the same time. Mice were individually identified (via ear tags) as part of normal facility procedure, allowing us to record individual (e.g. injury/wounds) as well as group (e.g. position within the cage) measures. The mice were kept in the same room within a Specific Pathogen-Free animal facility (Istituto di Ricerche Farmacologiche “Mario Negri”) with a regular 12:12h light/dark cycle (lights on 07:00 a.m.), at a constant room temperature range of 20–24°C, and relative humidity approximately 45–65%. The animal facility was monitored for pathogenic and opportunistic pathogens on a routine basis as for the FELASA recommendations/guidelines (quarterly and annual monitoring). All cages were changed every 14 days after the first week, inspected daily, and mice were provided with 100g/cage of the same bedding material (soft wood: Lignocel 3/4-S, J. Rettenmaier & Söhne GmbH + Co. KG, Rosenberg), ad libitum food (Global Diet 2018S, Harlan Italy, S. Pietro al Natisone, Italy) and water. At the end of the study all mice were euthanased by exposure to CO_2_ according to institutional protocol. Euthanasia was performed in a dedicated box/chamber with CO_2_, using a gradual 20% vol/min displacement rate. Procedures involving animals and their care were conducted in conformity with the institutional guidelines at the Mario Negri Institute in compliance with national (Decreto Legge nr 116/92, Gazzetta Ufficiale, supplement 40, February 18, 1992; Circolare nr 8, Gazzetta Ufficiale, July 14, 1994) and international laws and policies (EEC Council Directive 2010/63/EU 2010; Guide for the Care and Use of Laboratory Animals, U.S. National Research Council (Eighth Edition) 2011). The animal experiment described here was reviewed and approved by the Mario Negri Animal Care and Use Committee (IACUC) that includes members "ad hoc" for ethical issues.

### Experimental design

**Fig 1:**
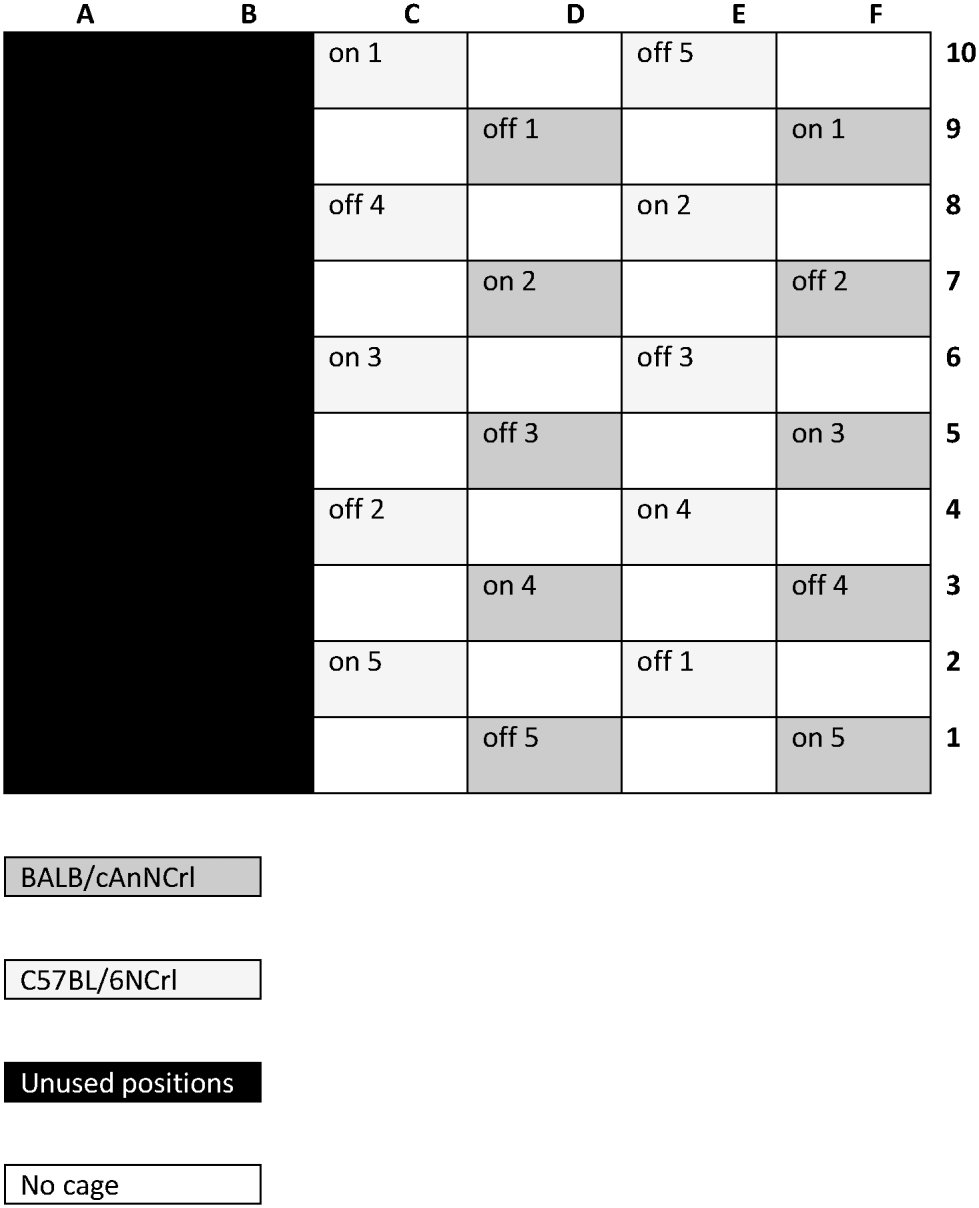
Representation of the cage allocation in the rack. EMF ‘on’ and ‘off’ cages are separated by empty spaces, with cage position balanced as far as possible for strain and treatment.

Mice were randomly allocated to IVC (individually ventilated) cages (cage model GM500, usable surface 500cm^2^, overall dimensions (W x D x H): 391 x 199 x 160 mm, ventilated in positive mode at 75 air changes per hour with a differential rate of -20% of the air exhausted, Tecniplast S.p.A., Buguggiate (VA) Italy) in single-strain groups of four individuals. Each cage position was fitted with an integrated EMF plate (DVC^™^ plate, Tecniplast SpA).with a potential frequency range of 5-100Hz, but with either the EMF plate turned ‘on’ (experimental group, n = 5 cages/strain) or turned ‘off’ (control group, n = 5 cages/strain). We measured the strength of the EMF in the specified range (5-100Hz), by placing the antenna of an electric and magnetic field analyser (model EHP-50, Narda) into the centre of each cage, close to the floor, when all the designated EMF ‘on’ plates were turned on. The average value recorded in the EMF ‘on’ cages at this point was 8.56V/m as r.m.s (+/- 0.77 SE) and 4.99V/m r.m.s. (+/- 0.32 SE) in the EMF ‘off’ cages. The cages were positioned within a cage rack modified to accommodate EMF plates such that cages were distributed evenly throughout the rack for both strain (C57BL/6NCrl and BALB/cAnNCrl) and condition (i.e. EMF ‘on’/’off’) to avoid any positional bias (see Fig 1), and with empty spaces in the rack surrounding each individual cage, to limit any potential carry-over between cages. Statistical analysis comparing EMF between cages (i.e. ‘on’ vs. ‘off’) confirmed that the EMF for cages that were ‘on’ was significantly higher (t18 = -4.299, P<0.001, Cohen’s *d* = 1.92). Apart from the EMF plates being turned ‘on’ or ‘off’, all other aspects of the cage environment was the same for all the animals.

### Experimental protocol

Measurements for each cage were collected over a seven week period, with an acclimatisation week (wk0) prior to the start of the six week experimental phase (wks 1–6). For those measures (e.g. bodyweight) that were taken regularly, the mice were assessed every week. Those measures that could only be taken once (e.g. tests of anxiety-related behaviour) were only tested at the end of the study. This allowed us to identify both short and longer-term responses to the experimental conditions, and this was also reflected in the choice of welfare indicators utilised.

### Measures of anxiety-related behaviour and welfare

Measures were selected to reveal any underlying differences in behaviour, cognition, and welfare (both short and longer-term), with an emphasis on those measures that could be simply, quickly and reliably recorded. There was therefore a focus on indirect measures of behaviour (e.g. injury scores as a reflection of aggression), ‘challenge’ tests that took place outside of the home cage (e.g. tests of anxiety-related behaviour) and unambiguous behavioural observations (e.g. position of mice within the cage).

#### General measures

Food (g) and water utilisation (g) were recorded on a weekly basis throughout the study. As this was determined by weighing food and water bottles, changes in the amount utilised could not solely be attributed to consumption.

#### Indirect behavioural and physical measures

These included: injury/wound scores [present/absent]; barbering score [present/absent]; whisker-trimming (whisker area only) score [present/absent] and bodyweight (g). These observations were made weekly for every individual in each cage, with a final observation taking place at the end of the study prior to euthanasia. Cages were observed in the same order, alternating between treatment cages.

#### Within-cage behavioural measure

**Fig 2:**
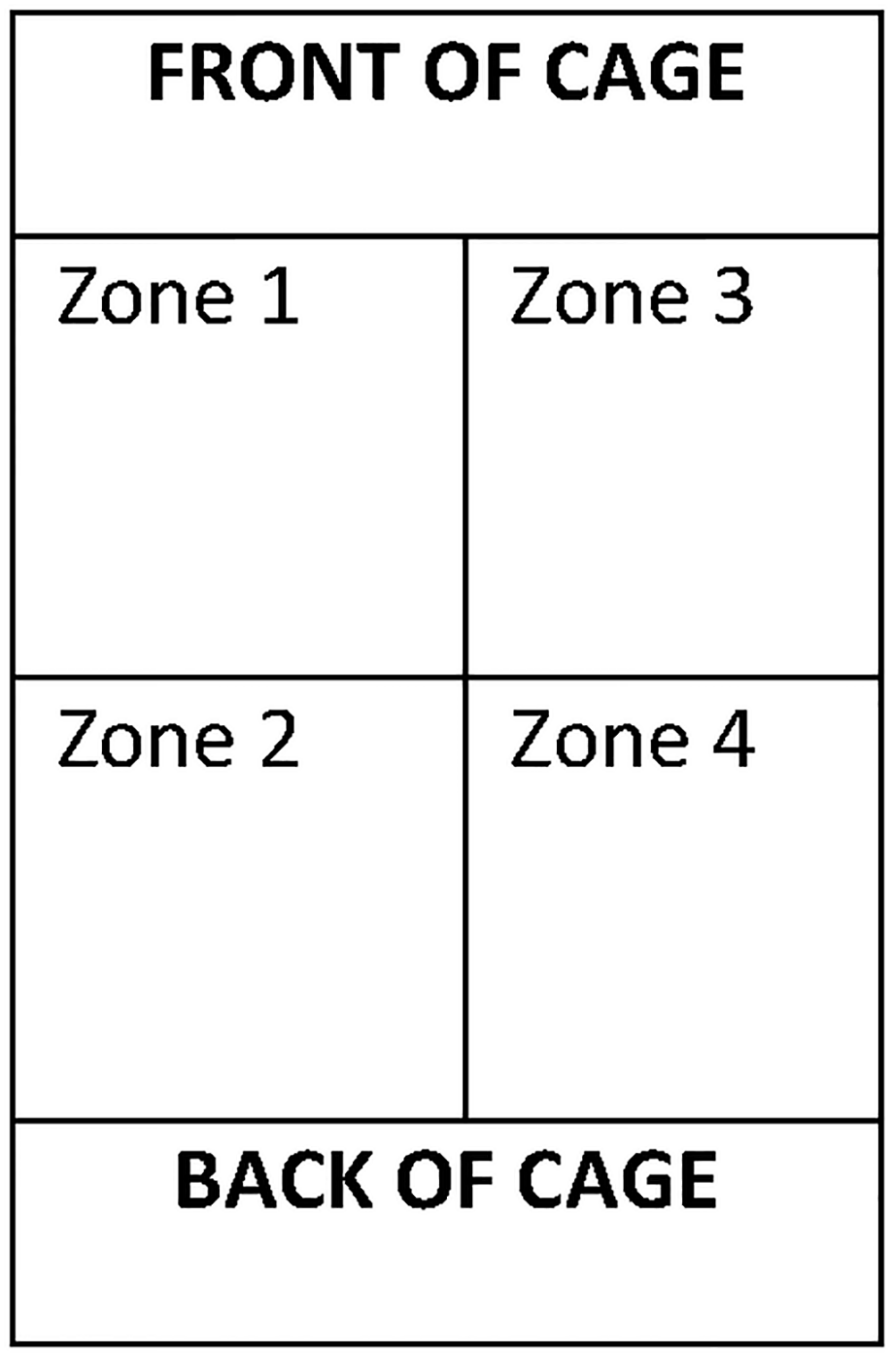
This figure shows how each cage was visually divided into zones of equal size.

Variation in spatial use of the home-cage may relate to a behavioural response to disturbance and/or aversive stimuli [15]. In order to determine how mice were spatially located within each cage (i.e. distributed evenly within the cage or located close together), each cage was visually ‘divided’ into zones (front left quarter/front right quarter/back left quarter/back right quarter, see Fig 2), and the number of mice in the different zones of each cage recorded. A mouse was determined as being in one zone of the cage when the majority (>50%) of the animal was in a particular zone, or the zone in which its head was located if it was divided equally (50%) between two zones. Weekly observations were carried out at the same time each week, alternating between treatment cages.

#### Test of anxiety-related behavior

At the end of the experiment, mice were tested in an open field test [a square grey Perspex arena (40 × 40 cm), 30 cm high, with the floor divided into 25 squares by black lines], tested under dim illumination provided by a 60 W lamp placed 1 m above the apparatus and pointed towards the ceiling. The open field tests were all performed on the same day (Day 1) from 0800:1800, with test order randomised for treatment/strain. The arena was placed in a specific room dedicated to behavioural analysis and separated from the room in which the mice were housed. All animals were placed in the centre of the empty arena and their behaviour video-recorded for 5 min. Mice were counted as being in a particular area (i.e. centre vs. external) when all four of its legs were positioned within the area, and the apparatus was wiped with 70% ethanol and dried prior to each test. The order in which animals were tested was alternated between treatments. The number of internal (the nine central squares) and external (the sixteen peripheral squares) squares crossed, the time spent in the central area of the open field (the nine central squares), the total number of rears, and total number of crossings were scored from video by experienced researchers ‘blind’ to the housing system as measures of general activity and anxiety-related behaviour (e.g. [15]). An additional outcome of this test was that it also habituated the subjects to the test arena in preparation for the subsequent cognitive testing.

#### Cognitive test

The novel-object recognition (NOR) test is a memory test that relies on spontaneous animal behaviour without the need for stressful elements, such as food or water deprivation or electric footshock (e.g. [16, 17]). The NOR test was performed across three consecutive days, from 0800:1800, with test order randomised for treatment/strain. In the NOR test, the mice were first familiarised with the test arena (Day 1 –which was the open field test), then introduced (Day 2) into the now familiar arena containing two identical novel objects that they could explore freely (the ‘acquisition phase’). The following objects were selected for use at random: a black plastic cylinder (4 × 5 cm), a glass vial with a white cup (3 × 6 cm), and a metal cube (3 × 5 cm). Twenty-four hours later (Day 3), the mice were placed back into the arena containing two objects (the ‘retention phase’), with one of the objects being the same as presented previously (the familiar object) and a new, different, one (the novel object), with object location balanced between subjects. Time spent investigating the two objects was video-recorded for 10 min for later scoring by two investigators blinded to the treatment. Results were expressed as a discrimination index (DI) (e.g. [18]), i.e., (seconds spent investigating the novel object − seconds spent investigating the familiar object) / (total time spent investigating both objects). Animals with no memory impairment should spend longer investigating the novel object in the test phase, giving a higher DI.

### Data analysis

In our experimental design individuals were only exposed to one of the two treatment groups (EMF ‘on’/’off’). The behaviour of individuals within a cage could be considered to be non-independent, so we used ‘cage’ as our experimental unit for all measures, combining data from all four mice within each cage to give a cage average. Where data met the requirements of parametric statistics (e.g. normal distribution of residuals), we used a univariate repeated measures General Linear Model (GLM) when testing measures recorded more than once (e.g. bodyweight), with Treatment (EMF ‘on’/’off’) and Strain (C57BL/6NCrl and BALB/cAnNCrl) as between-subject factors and Time as a within-subjects factor. For measures only taken at one time point (e.g. tests of anxiety-related behaviour) we used a univariate GLM with Treatment and Strain as between-subject factors. As spatial location within each cage was compositional data, it was tested in R using the package ‘Compositions’ with Treatment (EMF ‘on’/‘off’) and Strain (C57BL/6NCrl and BALB/cAnNCrl) as between–subject factors. For all analyses, if significant interactions were found, then related main factor results were not presented, and post-hoc investigation of interactions were carried out using either independent t-tests (e.g. comparison between strains at specific time points) or a repeated measures GLM (e.g. comparison between time points for a specific strain). Significance was taken as P<0.05, with Bonferroni adjustment for multiple testing made for post-hoc comparisons (adjusted p-values reported in text). All Treatment results are reported, regardless of statistical significance, as the main comparison of interest, whereas only significant effects are reported for other factors and interactions. With the exception of the compositional analysis, the statistical package used was SPSS (version 22).

## Results

### General measures

**Fig 3:**
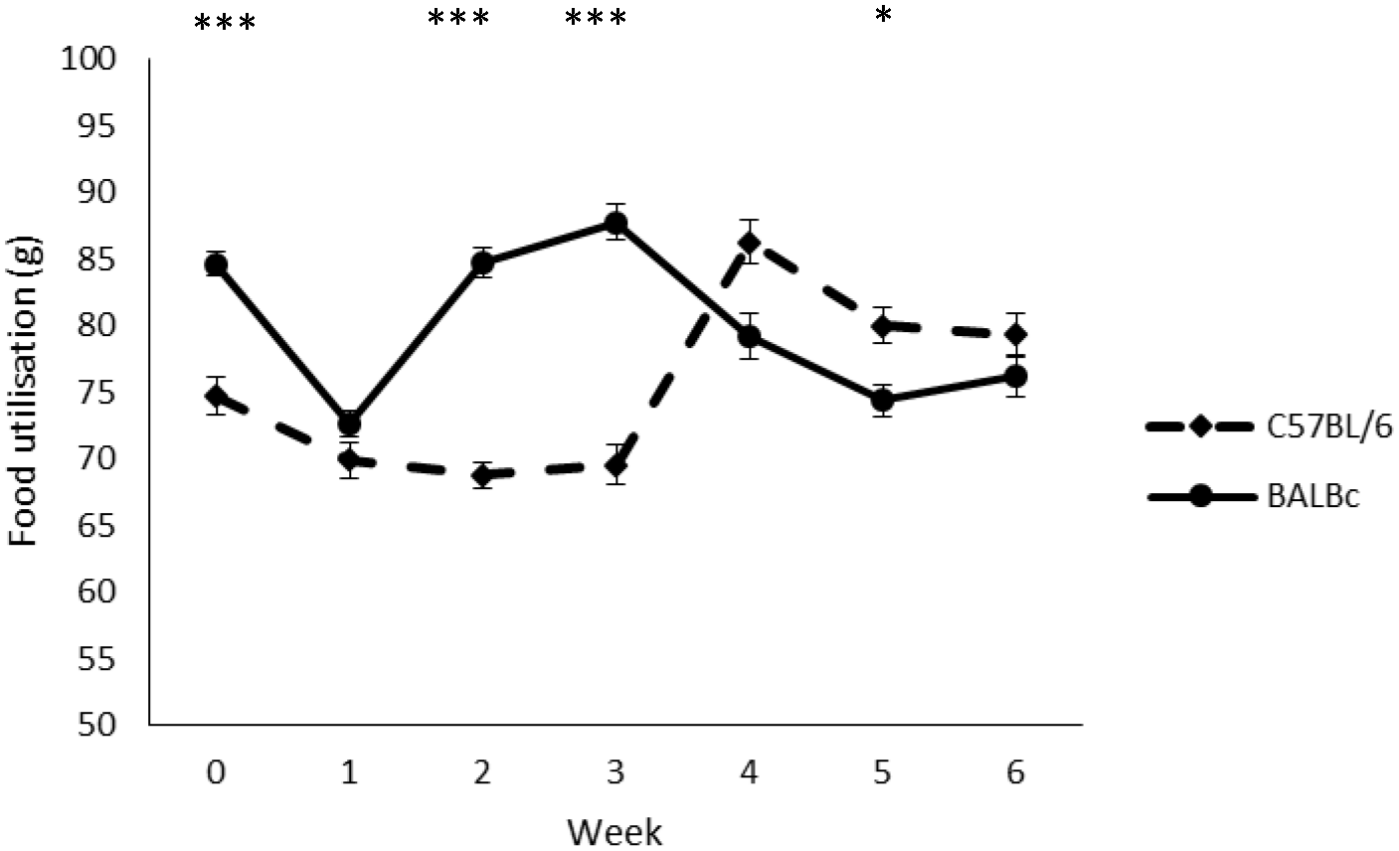
Change in food utilisation (g) over time (weeks) for the two strains of mouse (C57BL/6NCrl and BALB/cAnNCrl). Data (n = 10 cages/strain) are presented as means +/- standard error. ***P<0.001, *P<0.05.

There was no effect of Treatment (F1,16 = 0.051, P = 0.825, eta = 0.003, OP = 0.055) on food utilisation (mean±SE: ‘on’ 77.5±1.05; ‘off’ 77.9±1.05), but there was a significant Strain*Week interaction (Huynh-Feldt correction for non-spherical data F5.41,86.61 = 53.588, P<0.001, eta = 0.77, OP = 1.0), revealing that BALB/cAnNCrl mice used more food in weeks 0 (t18 = 6.061, adjusted P<0.001), 2 (t18 = 10.787, adjusted P<0.001) and 3 (t18 = 9.37, adjusted P<0.001) than C57BL/6NCrl mice, but less food in week 5 (t18 = -3.098, adjusted P = 0.043), with no differences at weeks 1, 4 and 6. Comparisons for each strain separately across time indicated significant differences between weeks for both BALB/cAnNCrl (Huynh-Feldt correction for non-spherical data F4.57,41.14 = 42.379, adjusted P<0.001, eta = 0.825, OP = 1.0) and C57BL/6NCrl (F6,54 = 39.308, adjusted P<0.001, eta = 0.814, OP = 1.0) mice, with BALB/cAnNCrl mice showing a reduction in food utilisation from week 3 onwards and C57BL/6NCrl mice showing a significant rise in week 4, and both strains showing an initial reduction in food utilisation from week 0 to week 1 (see Fig 3).

**Fig 4:**
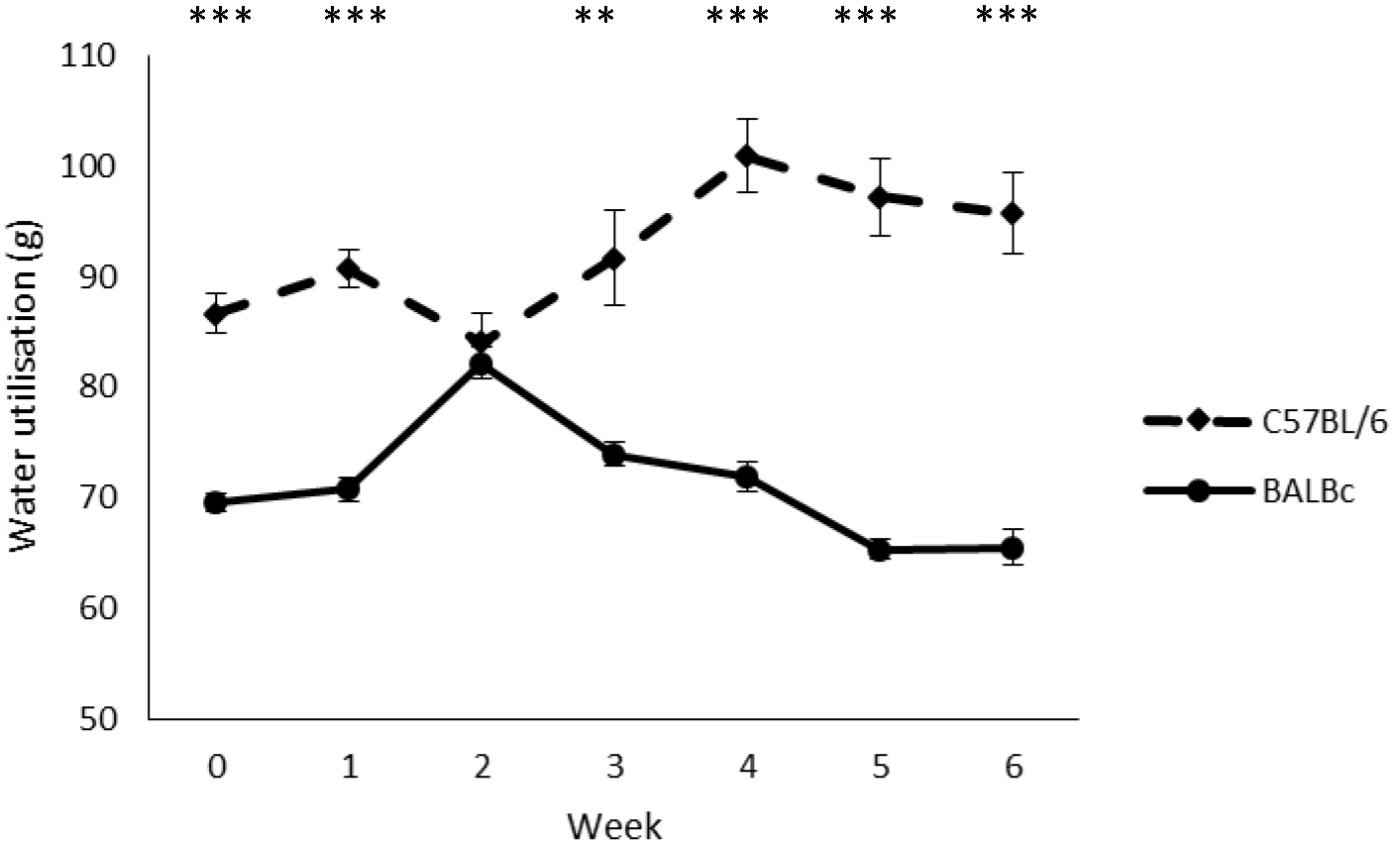
Change in water utilisation (g) over time (weeks) for the two strains of mouse (C57BL/6NCrl and BALB/cAnNCrl). Data (n = 10 cages/strain) are presented as means +/- standard error. ***P<0.001, **P<0.01.

There was no significant difference in water utilisation (mean±SE: ‘on’ 82.2±2.2; ‘off’ 81.5±2.2) between the treatments (F1,16 = 0.048, P = 0.83, eta = 0.003, OP = 0.055), but there was a significant Strain*Week interaction (Huynh-Feldt correction for non-spherical data F2.83,45.25 = 35.453, P<0.001, eta = 0.689, OP = 1.0) revealing that BALB/cAnNCrl mice used less water than C57BL/6NCrl mice for all weeks except week 2 (week 0: t18 = -8.712, adjusted P<0.001; week 1: t18 = -9.674, adjusted P<0.001; week 3: t18 = -4.057, adjusted P = 0.005; week 4: t18 = -8.143, adjusted P<0.001; week 5: t18 = -8.759, adjusted P<0.001; week 6: t18 = -7.618, adjusted P<0.001). Comparisons for each strain separately across time indicated significant differences between weeks for both BALB/cAnNCrl (F6,54 = 58.565, adjusted P<0.001, eta = 0.867, OP = 1.0) and C57BL/6NCrl (Huynh-Feldt correction for non-spherical data F2.26,20.32 = 15.239, adjusted P<0.001, eta = 0.629, OP = 0.999) mice, with BALB/cAnNCrl mice showing an increase in water use until week 2, before declining for the remainder of the study, and C57BL/6NCrl mice showing a rise until week 4 (see Fig 4).

### Indirect behavioural and physical measures

**Fig 5:**
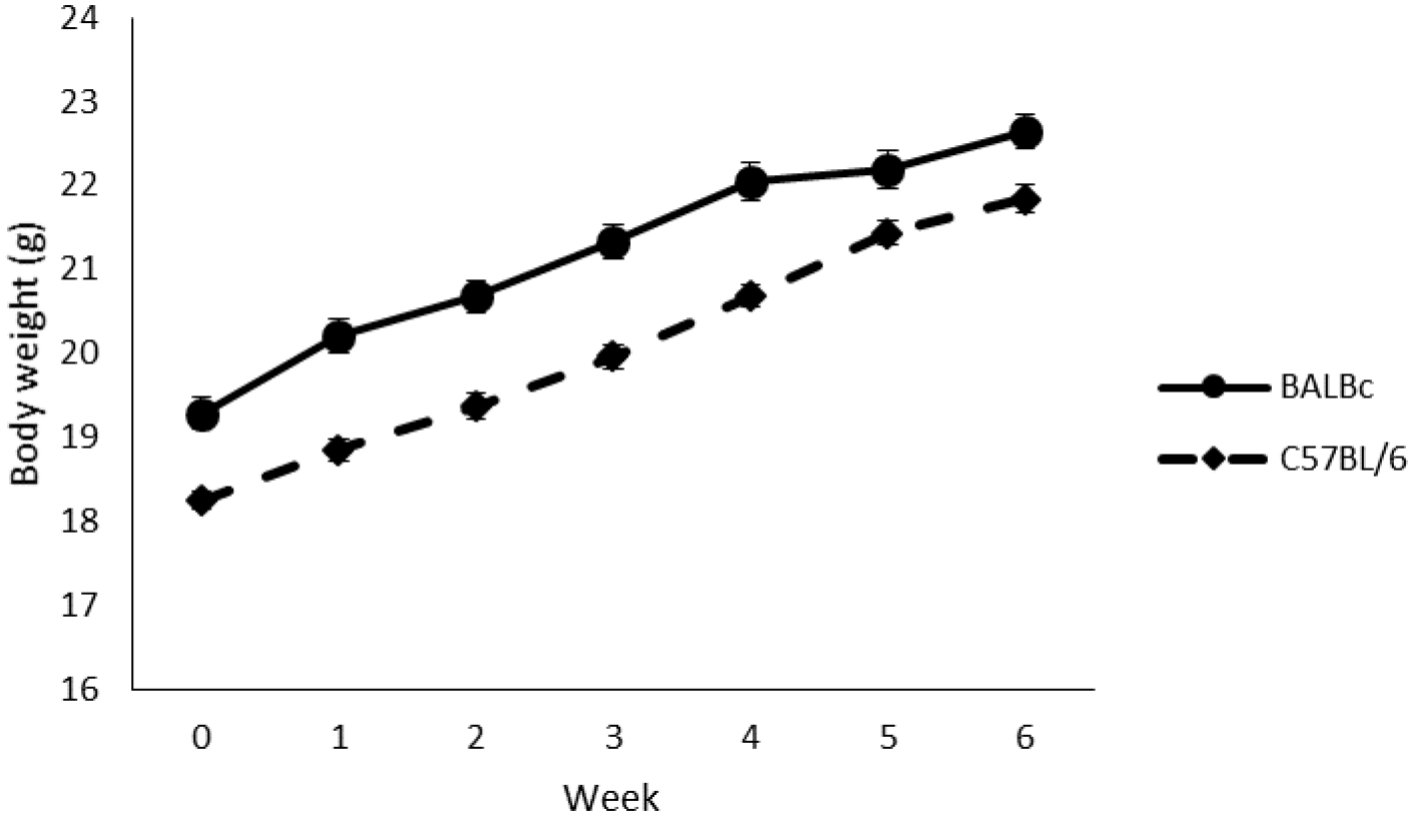
Change in body weight (g) over time (weeks) for the two strains of mouse (C57BL/6NCrl and BALB/cAnNCrl). Data (n = 10 cages/strain) are presented as means +/- standard error. All weeks P<0.001, except weeks 5 and 6 (no significant difference).

There was only one observation of injury and barbering being present, and that was in the same individual (a BALB/cAnNCrl mouse) from the EMF ‘off’ condition. No occurrences of whisker trimming were observed, and so no analyses for injury/barbering/whisker trimming were carried out. There was no effect of Treatment (mean±SE: ‘on’ 20.7±0.17; ‘off’ 20.6±0.17) on bodyweight (F1,16 = 0.021, P = 0.886, eta = 0.001, OP = 0.052), but there was a significant Strain*Week interaction (F6,96 = 7.442, P<0.001, eta = 0.317, OP = 1.0) revealing that BALB/cAnNCrl mice were heavier than C57BL/6NCrl mice at all time points except weeks 5 and 6 (wk0: t18 = 4.675, adjusted P = 0.0013; wk1: t18 = 5.516, adjusted P<0.001; wk2: t18 = 5.551, adjusted P<0.001; wk3: t18 = 5.668, adjusted P<0.001; wk4: t18 = 5.057, adjusted P<0.001), and that both strains increased in bodyweight over the course of the study (BALB/cAnNCrl: F6,54 = 284.486, P<0.001; C57BL/6NCrl: F5,64 = 346.958, P<0.001) (see Fig 5).

### Within-cage behavioural measures

**Fig 6:**
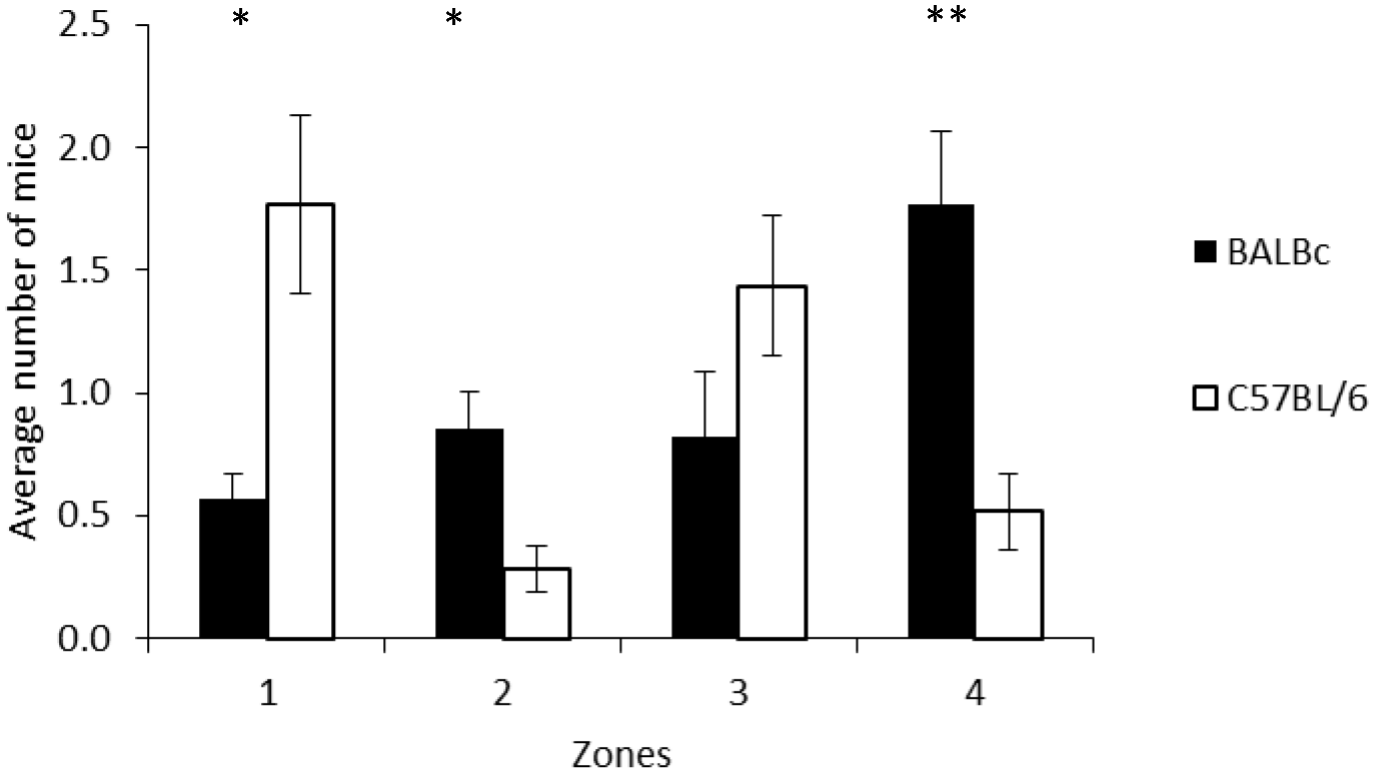
The average number of mice observed in each zone (1–4) of the cage for the two strains of mouse (C57BL/6NCrl and BALB/cAnNCrl). Data (n = 10 cages/strain) are presented as means +/- standard error. **P<0.01, *P<0.05.

Data for the spatial position in the cage were averaged for the different time points and compared for Treatment and Strain differences. There was no effect of Treatment (F1,16 = 0.1843, P = 0.9053, mean±SE: ‘on’ 1.0±0.2; ‘off’ 1.0±0.2), but there was a significant effect of Strain (F1,16 = 3.8032, P = 0.0348). Post-hoc investigation revealed that the two mouse strains differed in which zones they were typically positioned, with BALB/cAnNCrl mice compared to C57BL/6NCrl mice more frequently located in zones 2 (t18 = -3.142, adjusted P = 0.024) and 4 (t18 = -3.757, adjusted P = 0.004)–both at the back of the cage, and C57BL/6NCrl mice more frequently located in zone 1 (t18 = 3.181, adjusted P = 0.02)–at the front of the cage -than BALB/cAnNCrl mice (see Fig 6).

### Test of anxiety-related behaviour

**Fig 7:**
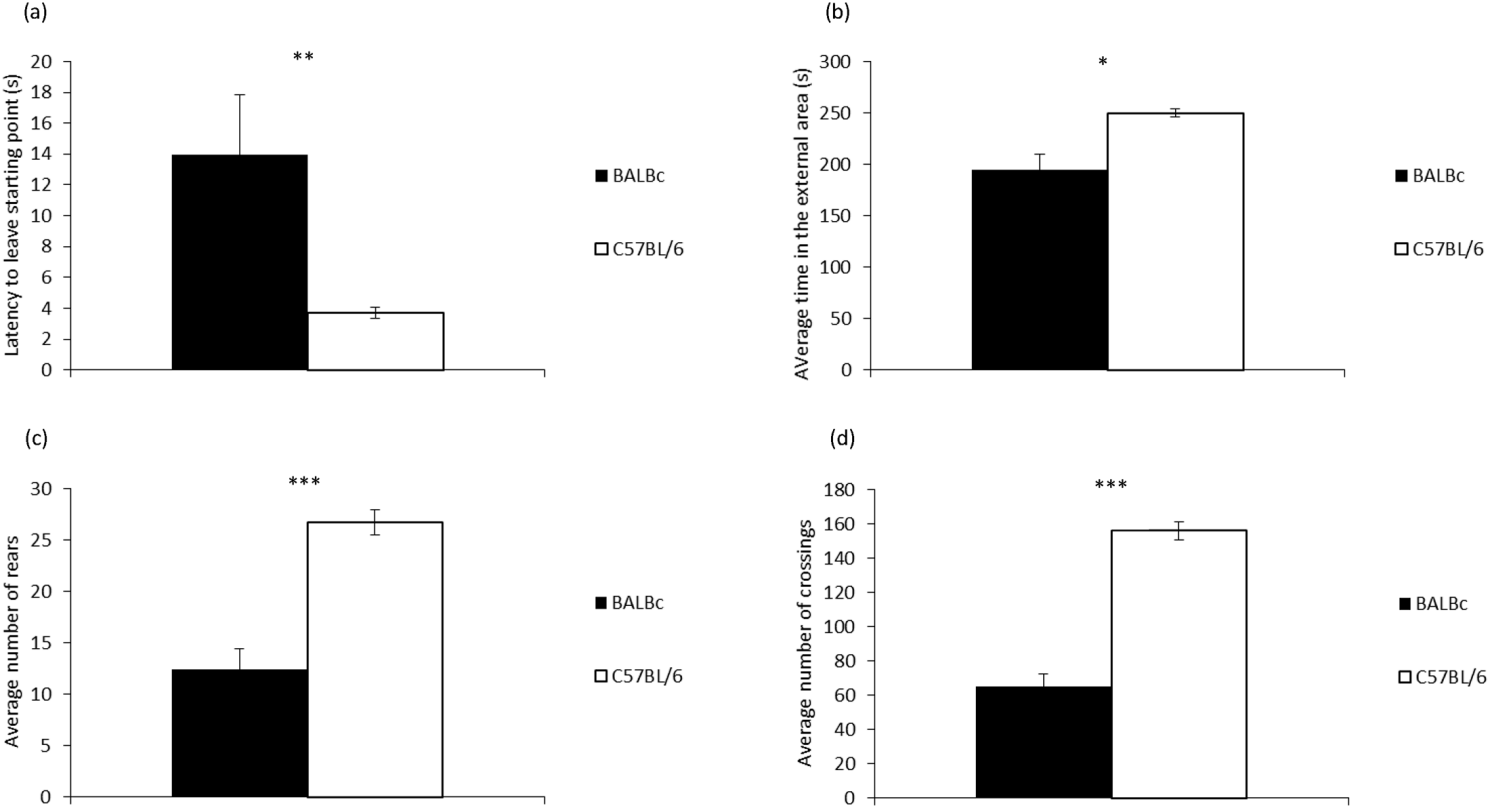
Differences between the two mouse strains (C57BL/6NCrl and BALB/cAnNCrl) in the open field test for (a) latency to leave the starting point; (b) time spent in the external area; (c) number of rears; and (d) number of crossings. Data (n = 10 cages/strain) are presented as means +/- standard error. ***P<0.001, **P<0.01, *P<0.05.

There were no significant effects of Treatment for any of the measures selected, either as a main effect (latency to leave the starting point: F1,16 = 1.685, P = 0.213, eta = 0.095, OP = 0.231, mean±SE: ‘on’ 6.6±2.4; ‘off’ 11.1±2.4; time spent in the external area: F1,16<0.001, P = 0.994, eta<0.001, OP = 0.05, mean±SE: ‘on’ 222.3±13.5; ‘off’ 222.5±13.5; total rearing: F1,16 = 0.96, P = 0.342, eta = 0.057, OP = 0.152, mean±SE: ‘on’ 20.9±2.0; ‘off’ 18.2±2.0; total crossings: F1,16 = 0.371, P = 0.551, eta = 0.023, OP = 0.088, mean±SE: ‘on’ 113.8±7.7; ‘off’ 107.2±7.7) or interactions (latency to leave the starting point: F1,16 = 0.973, P = 0.339, eta = 0.057, OP = 0.153; time spent in the external area: F1,16 = 0.002, P = 0.969, eta<0.001, OP = 0.05; total rearing: F1,16 = 0.795, P = 0.366, eta = 0.047, OP = 0.134; total crossings: F1,16 = 0.572, P = 0.46, eta = 0.035, OP = 0.11). Significant Strain effects were identified for all measures, as follows: BALB/cAnNCrl mice were slower to leave the starting point (centre) (F1,16 = 8.827, P = 0.009, eta = 0.356, OP = 0.796), spent less time in the external area (F1,16 = 8.301, P = 0.011, eta = 0.342, OP = 0.772), reared less (F1,16 = 25.4, P<0.001, eta = 0.614, OP = 0.997) and carried out significantly less crossings (F1,16 = 70.56, P<0.001, eta = 0.815, OP = 1.0) than the C57BL/6NCrl mice (see Fig 7(a)–7(d)).

### Cognitive test—NOR test

For the NOR test we used a criterion that, in order to be included in the analysis, mice had to investigate *both* objects in *both* phases of the test (i.e. in the ‘acquisition’ and the ‘retention’ phase)–otherwise it could not be reliably inferred that they had both acquired and remembered the identity of the stimuli. All of the C57BL/6NCrl mice achieved this criterion, whereas 16/40 BALB/cAnNCrl mice were excluded (7 from the EMF ‘ON’ and 9 from the EMF ‘OFF’ conditions). Because of this high level of exclusion, the BALB/cAnNCrl data was not analysed further. A comparison of the C57BL/6NCrl mice between the EMF ‘on’ and ‘off’ treatments revealed no statistically significant difference (t8 = -1.119, P = 0.296. Cohen’s *d* = 0.708, mean±SE: ‘on’ 0.04±0.07; ‘off’ 0.17±0.07).

## Discussion

Taking the results of this study together as a whole, we found no effect of continuous low frequency EMF, as an integrated part of a rodent housing system, on mouse behaviour and welfare. We did, however, observe clear differences between the two mouse strains studied (BALB/cAnNCrl and C57BL/6NCrl) for a variety of measures, ranging from bodyweight, water utilisation, and position within the cage to contrasting responses in the tests of anxiety-related behaviour (OF test) and cognition (NOR test).

The lack of an observed effect of EMF may be due to a number of reasons. Firstly, as a low frequency (range 5-100Hz) EMF, it may be that there is no significant influence on bodyweight, behaviour and welfare—even when experienced continuously for six weeks as an integrated part of the housing system. Previous studies investigating similarly low frequency EMFs, albeit not as an integrated part of the animal housing system, have found conflicting effects on anxiety-like behaviour, bodyweight, locomotion and cognition. For instance, in studies where EMF exposure was relatively short-term, neither four days [8] nor 200hrs [10] of 60Hz EMF exposure appeared to have had an effect on anxiety-like behaviour or memory retention, but did appear to reduce locomotion/mobility. In contrast, studies of slightly longer-term exposure, [19] (60Hz EMF for 30 days) and [4] (50Hz EMF exposure for 4hrs a day for up to 25 days) found no effect on locomotion, but [4] did observe increased anxiety-like behaviour of rats in OF and EPM tests and [20] (50Hz EMF for 30 days) found a gradual bodyweight loss. There are also contradictory results when it comes to determining EMF impact on cognition. [7] (50Hz EMF for 20min) found an impaired spatial memory retention, whereas [6] (60Hz EMF exposure for 2hrs/day for nine days) observed an improvement in social recognition. In contrast, [19] (60Hz EMF for 30 days) found an impairment in social recognition. Interestingly, when higher frequency (2.45GHz) EMFs are considered, for both short (45min: [3]) and long-term exposures (up to 120 days: [2]), there appeared to be no effect on various measures of anxiety and depression-like behaviour, suggesting that low and high frequency EMFs may affect mice in different ways. Taking these results together, there could be time-related effects of low EMF exposure, with short-term effects on locomotion/mobility giving way to potential impacts on anxiety-like behaviour with longer exposures alongside an unpredictable influence on cognitive performance—patterns not reflected in our own results. However, the apparent lack of effect following both short and long-term high frequency EMF exposure suggests that such variation in observed responses for low EMF studies may be more likely explained by differences in methodology between studies other than those relating to the frequency and/or duration of EMF exposure.

For instance, behavioural studies may choose to focus on anxiety and/or depression-like behaviour, locomotion/mobility, cognition or general ‘in-cage’ behavioural measures either in isolation or in combination. Even when studies share the same focus, such as anxiety-like behaviour, then this might be investigated using a variety of different tests [21], such as the open field test or elevated plus maze, and, even when the same test is selected (e.g. open field), then the design of the test (e.g. variation in dimensions, lighting, construction materials, starting position, test duration, variables recorded: [22, 23]) may have an influence on the results obtained beyond species/strain/establishment variation. Comparison between studies is difficult when so many factors vary, and it would make sense to ensure consistency between future studies to build a more robust overall picture of EMF effects. In our study, because we were studying a specific housing system with an integrated EMF exposure at a pre-determined frequency, the focus was on differences between the system with EMF ‘on’ vs. ‘off’ rather than the response to EMF per se. Although there were no significant differences in anxiety-related behaviour, bodyweight, object recognition and general behaviour, such results would need to be taken into consideration alongside the results of similar studies using alternative approaches, for instance, use of pathological indicators [24].

Confidence in the lack of a significant effect of EMF in the current study was boosted by finding clear, expected, strain differences across the range of recorded measures. For example, in the open field test the BALB/cAnNCrl mice were slower to leave the central starting square, showed less rearing behaviour and made fewer crossings compared to C57BL/6NCrl mice—showing evidence of increased anxiety-related behaviour and reduced locomotion/mobility. The BALB/cAnNCrl mice were also generally heavier in bodyweight, appeared to drink less (in terms of lower water utilisation) and were more often found at the back of the cage while C57BL/6NCrl mice were more likely to be located at the front of the cage. For the novel object recognition test, almost half of the BALB/cAnNCrl mice failed to reach the inclusion criterion whereas all the C57BL/6NCrl mice successfully achieved the criterion. Such strain differences in anxiety-related behaviour and bodyweight have been noted previously (e.g. [15, 25]). The differences in cage location and NOR performance may also be related to anxiety-related behaviour. Interestingly, [26] found few strain differences in the NOR test, and particularly noted the increased exploratory behaviour of the BALB/cAnNCrl mice compared to the C57BL/6NCrl mice—a result that appears to contrast with our own observations. But, in their study they included extensive pre-training; potentially rendering their NOR test less anxiogenic.

## Conclusions

In summary, our results indicate that, for the measures recorded, there was no significant impact on the behaviour and welfare of low frequency EMF exposure experienced continuously over a six-week period as an integrated part of this IVC housing system for BALB/cAnNCrl and C57BL/6NCrl mice. Housing systems that include appropriate low frequency EMF monitoring technology may therefore be suitable for use without influencing either animal welfare or scientific outcomes, opening up this technological advance for application to a range of new opportunities.

## Supporting information

S1 Data Raw data used during analyses. (XLS)

## Abbreviations

EMF: Electromagnetic field
GLM: General linear model
IVC: Individually ventilated cage
Ƞ: Effect size
NOR: Novel object recognition test
OF: Open field test
OP: Observed power
